# Association Between Vitamin D and Zinc Levels With Alopecia Areata Phenotypes at a Tertiary Care Center

**DOI:** 10.7759/cureus.14738

**Published:** 2021-04-28

**Authors:** Saeed M Alamoudi, Siham M Marghalani, Rakan S Alajmi, Yara E Aljefri, Abdullah F Alafif

**Affiliations:** 1 Medicine, King Saud Bin Abdulaziz University for Health Sciences, Jeddah, SAU; 2 Dermatology, King Abdulaziz Medical City, Western Region, Jeddah, SAU

**Keywords:** alopecia areata, comorbidities, vitamin d deficiency, zinc, saudi arabia

## Abstract

Objectives

Alopecia areata (AA) is a common immune-mediated hair disorder that presents in different clinical patterns. This study aims to find the association between vitamin D and zinc levels with AA phenotypes, to determine the common comorbidities in AA patients, and to assess the influence of age, gender, and body mass index (BMI) on AA phenotypes.

Methods

This is a cross-sectional study conducted at King Abdulaziz Medical City (KAMC) in Jeddah, Saudi Arabia. Data were collected through retrospective chart review of the electronic medical record system (BestCare) and by utilizing a structured data collection sheet.

Results

A total of 177 patients were clinically diagnosed with AA with a mean age of 28.37 ± 12.68 years. The mean vitamin D level was 49.14 ± 29.09 nmol/L. Zinc levels were reported in only 22 patients, among which, only one patient had deficient levels. The mean zinc level was 9.8 ± 1.5 µmol/L. Patchy alopecia areata (60.45%) was the most common phenotype followed by universalis (9%) and totalis (7%). Hypothyroidism (11.8%) was the most prevalent comorbidity followed by atopic diseases (10.7%), then both diabetes and mood disorders (6.2%).

Conclusion

Deficient serum vitamin D levels were present in 62.7% of patients with AA. Nevertheless, no statistically significant relation was detected between vitamin D status and patterns of alopecia areata (P=0.108). A limited number of our sample had records of zinc levels with a mean serum of 9.8 ± 1.5 µmol/L and only one patient was found to be deficient.

## Introduction

Alopecia areata (AA) is an autoimmune disorder mediated by T-cells that is characterized by transient hair loss and affects approximately 2% of the general population at some point in their lives [1]. The clinical expression of AA can either involve small localized areas of the scalp and beard (patchy AA), or the entire scalp (AA totalis), or it involves the entire body (AA universalis) [1,2]. Although dermoscopy and histopathology can support the diagnosis, AA is mainly diagnosed by its clinical manifestations [1]. Traditional therapeutic approaches to AA include corticosteroids, immune therapy, and phototherapy [3].

Vitamin D represents a group of fat-soluble steroids obtained by dietary intake or synthesized by epidermal keratinocytes in humans [4]. Enzymatic conversion into the active form 1,25-dihydroxyvitamin D (1,25-(OH)2D) is required for both forms [4]. Active vitamin D serves numerous roles, one of which is the variation of the immune response which, in some cases, is believed to be related to the activity and onset of autoimmune diseases [4-6]. It is estimated that vitamin D deficiency affects 41.2-100% of Saudi women of all ages [7]. On the other hand, vitamin D deficiency is estimated to be found among 32.5-92.6% of Saudi men [7]. One explanation for the widespread prevalence of vitamin D deficiency in the Saudi population, despite ample sunshine throughout the year, is related to the national dress code which prevents males and females from adequately exposing their skin to ultraviolet radiation required for sufficient synthesis of vitamin D [8]. Low vitamin D levels have been implicated in the pathogenesis of various autoimmune conditions such as psoriasis, rheumatoid arthritis, type 1 diabetes mellitus, and AA [4-6]. Vitamin D is theorized to have an immunomodulatory effect via its activity on various components of the immune system such as suppressing Th1 cells, enhancing Th2 and Tregs functions, altering dendritic cell activity, and inhibiting cytokines that stimulate Th17 [5,9]. Also, vitamin D deficiency was found to be more prevalent among patients with AA compared to other dermatological conditions such as vitiligo [5,10].

Zinc is a mineral that is crucial for the function of a variety of catalytic enzymes [11]. Copper/zinc superoxide dismutase and alkaline phosphatase are examples of enzymes that depend on zinc to facilitate their catalytic activity [11]. Moreover, it is linked to hair biology and metabolism through its immunomodulatory and antioxidant effects as well as inhibiting hair follicle endonucleases involved in keratinocyte apoptosis [12]. It is postulated that zinc deficiency is implicated in the pathogenesis of AA by dysregulation of copper/zinc superoxide dismutase with a net imbalance in oxidant/antioxidant activity [11,12]. Multiple studies have investigated levels of trace elements such as zinc in AA patients compared to healthy controls, but no studies explored the association between serum zinc levels and specific AA phenotypes.

Accumulating evidence in the literature suggests an inverse relationship between vitamin D and zinc levels with the severity of AA [4,5,13]. As there is a possible link between AA and vitamin D and zinc levels, both should be explored and investigated more comprehensively regarding potential therapeutic roles in ameliorating disease severity or monitoring response to treatment in patients with alopecia areata [10]. To the best of our knowledge, the relationship between vitamin D and zinc levels with phenotypes of AA has not been explored in Saudi Arabia. This study aimed to investigate the association between vitamin D and zinc levels and phenotypes of AA as well as common comorbid conditions found in patients with AA.

## Materials and methods

This cross-sectional study was conducted at King Abdulaziz Medical City (KAMC) in Jeddah, Saudi Arabia. Data were collected through a retrospective chart review of the electronic medical record system (BestCare) and by utilizing a structured data collection sheet. The data collection sheet consisted of socio-demographic variables like age, gender, and BMI. Moreover, clinical variables like alopecia areata type, vitamin D levels, AA disease activity, and comorbidities were assessed. Patients were considered to have a stable disease if their records revealed hair regrowth or no additional hair loss whereas active disease was defined as continuing hair loss despite treatment. Patients with vitamin D levels below 50 nmol/L were considered deficient while those with levels more than 125 nmol/L were labeled as having toxic vitamin D levels. With regards to zinc levels, a level between 10.7-22.9 µmol/L is considered normal, and a level below 7 µmol/L is considered deficient. The obtained data from the collection sheet was entered into Microsoft Excel 2016 (Microsoft Corporation, Redmond, WA, USA). Then entered and analyzed in IBM Statistical Package for the social sciences (SPSS) version 25.0 (IBM Corp., Armonk, NY, USA). Qualitative variables were presented using descriptive statistics in the form of categories and summarized as frequencies and percentages. Data comparison was interpreted using Chi-square test and Fisher exact test, and a p-value that is less than 0.05 was considered to be significant. All patients’ data is confidential and ethical approval was received from the Institutional Review Board at King Abdullah International Medical Research Centre, National Guard Health Affairs (NGHA), Jeddah, Saudi Arabia (reference number: JED-20-427780-162933) in accordance with The Code of Ethics of the World Medical Association (Declaration of Helsinki).

## Results

Of the total 177 participating alopecia areata patients, 85 (48%) were males and 92 (52%) were females. Regarding age, patients were grouped into three categories (<18 years, 18-40 years, >40 years). Patients' ages ranged from 2 to 79 years old. The mean age was 28.37 ± 12.68 years. The mean BMI was 26.44 ± 7.5 kg/m2, with the highest BMI being 59.9 kg/m2 and the lowest being 9.04 kg/m2. Normal BMI was reported in 81 (45.7%) patients, 39 (22%) patients were overweight, and 57 (32%) were obese. 

Patchy alopecia areata was the most common type (60.45%), followed by universalis (9%) and totalis (7%). The most commonly identified comorbidity was hypothyroidism (11.8%) followed by atopic diseases (10.7%), and both diabetes and mood disorders (6.2%). Regarding disease activity, 67 (37.8%) patients had active disease, 49 (27.7%) had stable disease, and disease activity was not reported in 61 (34.5%). Table 1 summarizes socio-demographic variables in association with alopecia areata type.

**Table 1:** Sociodemographic variables and alopecia areata phenotypes *Fisher exact test     ** Chi-square test

Variable	Alopecia Areata Types					P-Value
		Patchy	Universalis	Totalis	Unspecified	
Age	< 18 years	24	8	6	7	0.113*
	18-40 years	58	9	8	32	
	> 40 years	17	1	2	5	
Gender	-Male	50	7	8	20	0.712**
	-Female	59	9	5	19	
BMI	-Normal	48	9	8	18	0.972*
	-Overweight	23	4	3	9	
	-Obese	36	3	2	14	
Comorbidities	-No	62	9	11	3	0.131*
	- at least 1	44	44	2	2	

Zinc levels were reported and recorded only in 22 patients. The highest zinc level was 15.54 µmol/L and the lowest was 6.14 µmol/L with a mean zinc level of 9.8 ± 1.5 µmol/L. Out of the reported zinc levels, only one patient had a deficient zinc level (6.14 µmol/L). The mean vitamin D level was 49.14 ± 29.09 nmol/L. Vitamin D level was deficient in 110 (62%) patients, optimum in 47 (26.5%), and toxic in 20 (11.3%). The highest vitamin D level was 177.8 nmol/L and the lowest was 20.1 nmol/L. Refer to Table 2 for a better view of the association between vitamin D levels with alopecia areata type and disease activity. 

**Table 2:** The association between vitamin D levels and alopecia type and disease activity *Fisher exact test

Alopecia Type		Vitamin D level			P-value
		Insufficient/ deficient	Normal	Toxic	
	Patchy	64	32	11	0.108*
	Universalis	9	4	3	
	Totalis	7	2	4	
	Unspecified	30	9	2	
Disease Activity	Stable	29	16	4	0.398*
	Active	40	17	10	
	Unknown	42	13	6	

Mood disorders were present in 11 alopecia areata patients, of whom, six had a deficient vitamin D level, two had a normal vitamin D level, and three had a toxic vitamin D level. There was no significant association between vitamin D levels and mood disorders (P=0.195). 

## Discussion

Alopecia areata is an autoimmune disorder that appears in different clinical patterns such as patchy, universalis, and totalis. Our study revealed that patchy AA was the most common phenotype and hypothyroidism was the most common comorbidity in AA patients. No association was discovered between vitamin D and zinc levels with AA phenotypes. Deficient vitamin D levels were noted in approximately 62% of our sample and no significant association with psychological disorders was observed. 

An epidemiological local study conducted at a tertiary care center in Jeddah demonstrated that the highest prevalent subtype among 216 alopecic patients was also patchy alopecia (6.48%), then totalis (4.63%) and universalis (1.85%) [14]. Similarly, a study conducted in Egypt which included 60 patients stated that patchy alopecia was the commonest (53.3%), then both totalis and universalis with lesser frequencies (26.7%) [15]. Besides, a study in Tunisia performed on 204 patients showed similar findings to ours [16]. Therefore, patchy alopecia is the most commonly encountered pattern of hair loss in dermatology clinics as revealed by our study and other previous studies.

In the present study, we assessed vitamin D levels in relation to the pattern or extent of hair loss in patients clinically diagnosed with AA. The difference observed between serum vitamin D levels and patterns of AA was not statistically significant (P=0.108). Our results were supported by a previous study conducted in Turkey which also did not find any association between vitamin D concentrations and the different patterns of A A[17]. In contrast to our results, a study involving 86 alopecic patients suggested the role of vitamin D in the pathogenesis of AA [5]. Likewise, a significant relation was reported by another study conducted in Egypt and one conducted in India [15,18]. Despite an established biological association between vitamin D levels and AA, evidence from clinical studies shows conflicting results. This recognizable inconsistency in results between studies can be explained by multiple factors such as having an unequal number of examined patients in each study, different clinical data, methodological variations, seasonal variability in serum vitamin D levels, and different geographical areas [19].

Accumulating evidence in the literature suggests that psychological disorders are positively linked to AA [20,21]. The nature of this relationship has been described as “Bidirectional” since psychiatric disorders can trigger or exacerbate AA [21]. On the other hand, AA can negatively influence self-image and quality of life and thus lead to psychological disorders [21]. We found that 6.2% of our sample had a diagnosed psychiatric disorder such as depression or anxiety. Other retrospective cross-sectional studies found a higher prevalence of depression and anxiety in tertiary care facilities over 11 years, reaching 25% of all AA patients [22,23]. Several studies have shown that AA patients had higher levels of mood disorders compared to their age-matched controls as well as lower self-esteem, poorer quality of life, and self-image [20]. Hair loss localized to the eyebrows or eyelashes among AA patients was significantly associated with higher rates of depression and anxiety [21]. Overall, it is clear that the psychological impact of AA is profound and should be taken into consideration when caring for these patients.

Furthermore, our study revealed that there is no significant association between vitamin D deficiency and having mood disorders in patients with alopecia areata. This is in contrast to other studies that found a significant association between deficient vitamin D levels in patients with affective disorders compared to healthy age-matched controls [24]. Low vitamin D levels have been linked to an increased occurrence of mood disorders, yet this association has not been thoroughly investigated in AA patients as compared to other medical conditions [25]. Moreover, a study assessing vitamin D levels in patients with fibromyalgia demonstrated that high levels of depression and anxiety were strongly related to vitamin D deficiency [26]. Another study among women in early pregnancy suggested that vitamin D levels may be related to symptoms of depression [27]. All of this brings into question whether having mood disorders in patients with alopecia areata is related to deficient vitamin D levels independent from other factors such as sunlight exposure, physical activity, and poor diet.

**Figure 1:**
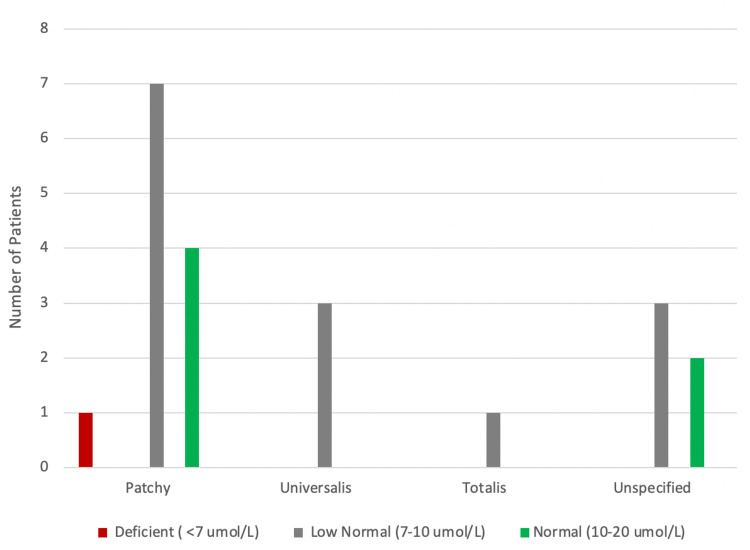
Zinc levels in relation to alopecia types

In addition, measuring zinc levels is not a routine test to be done in patients with AA and this is why only 22 of our patients had zinc level results with a mean of 9.8 ± 1.5 µmol/L. Among those 22 patients, only one had deficient zinc levels. Moreover, the majority of our sample (14 out of 22) had serum zinc levels in the low normal range (7-10 µmol/L) and only one patient was deficient as demonstrated in Figure 1. 

In comparison to healthy age-matched controls, several studies demonstrated that patients with AA have markedly lower mean serum zinc levels [12,28]. For example, a meta-analysis that explored the association between serum trace element levels and AA found that patients with AA have deficient zinc and selenium levels, which might suggest increased susceptibility of developing AA [28]. In addition, zinc has been suggested as an adjuvant therapy for AA patients with low serum zinc considering its role in immunomodulation and hair metabolism [29] However, a double-blind trial evaluating the effect of oral zinc supplementation among AA patients found no effect compared to placebo [30]. In summary, AA patients have been shown to have significantly lower zinc levels yet the association between zinc levels and specific AA phenotypes requires further investigation.

Among the population investigated in our study, we found that thyroid disorders, particularly hypothyroidism, were the most common comorbidity in patients with AA followed by a personal history of atopic disease which accounted for 11.8% and 10.7%, respectively. This is in contrast with other studies in the literature which demonstrated atopic disease as the most common comorbidity followed by hypothyroidism as the second most common. For example, Arousse et al. found that atopic disease occurred in 18.1% of their sample whereas hypothyroidism was noted in 12.7% of cases [16]. However, a previous local study demonstrated findings similar to ours as hypothyroidism was the most common comorbidity in their sample [14]. It should be noted that hypothyroidism was more prevalent among AA patients than any singly atopic entity, yet atopic disease, in general, was more common [14]. Moreover, a systematic review and meta-analysis which included 87 studies found that patients with AA had significantly higher odds of having atopic disease [4]. Our study showed similar findings with regards to diabetes in patients with AA as only 6.2% of cases had a diagnosis of diabetes, whether type I or type II. Similarly, Alshahrani et al. observed that 5% of their AA cases had comorbid diabetes [14]. Also, meta-analyses showed that patients with AA had lower odds of developing diabetes [4].

This study had some limitations mainly due to its small sample size. Moreover, our study was retrospective and conducted at a single center, making it difficult to generalize the results. We recommend doing prospective multicenter studies with larger sample sizes to investigate the outcome. Furthermore, vitamin D deficiency is common among our population and its effect on disease severity requires additional exploration. Further clinical studies with larger samples are warranted to better delineate whether or not vitamin D and serum zinc levels influence the type or course of AA. 

## Conclusions

No significant association was found between vitamin D levels and AA phenotype or disease activity. The majority of patients had deficient vitamin D levels (62%). The most common phenotype of AA is patchy (60.45%) followed by universalis (9%) then totalis (7%). The most common comorbid conditions in patients with AA were hypothyroidism (11.8%) followed by atopic disease (10.7%). Only 22 patients with AA had records of zinc levels and only one had deficient zinc levels. Vitamin D deficiency is common among our population and its effect on disease severity requires additional exploration. Further clinical studies with larger samples are warranted to better delineate whether or not vitamin D and serum zinc levels influence the type or course of AA.
